# Chemical Composition, Antioxidant, and Antimicrobial Activities of Leaves of *Ajuga Iva*

**DOI:** 10.3390/molecules27207102

**Published:** 2022-10-20

**Authors:** Hajer Ammar, Imen Touihri, Ahmed Eid Kholif, Yassine M’Rabet, Rym Jaouadi, Mireille Chahine, Mario E. de Haro Marti, Einar Vargas-Bello-Pérez, Karim Hosni

**Affiliations:** 1 Laboratoire de Systèmes de Production Agricole et Développement Durable “SPADD”, University of Carthage, Ecole Supérieure d’Agriculture de Mograne, Mograne Zaghouan 1121, Tunisia; 2 Laboratoire des Substances Naturelles, Institut National de Recherche et d’Analyse Physico-Chimique (INRAP), Biotechpôle de Sidi Thabet, Ariana 2020, Tunisia; 3 Dairy Science Department, National Research Centre, 33 Bohouth St. Dokki, Giza 12622, Egypt; 4 Department of Animal, Veterinary and Food Sciences, University of Idaho, 315 Falls Ave, Twin Falls, ID 83301, USA; 5 Gooding County Extension, University of Idaho, 203 Lucy Lane, Gooding, ID 83330, USA; 6 School of Agriculture, Policy and Development New Agriculture Building, University of Reading, Earley Gate Whiteknights Road, P.O. Box 237, Reading RG6 6EU, Berkshire, UK

**Keywords:** *Ajuga iva* L., antimicrobial activity, antioxidant activity, fatty acids, HPLC-PDA-ESI-MS/MS, phenolics, essential oil

## Abstract

The main objective of this research was to study the biological characteristics in terms of antioxidant and antimicrobial activities of *Ajuga iva* and determine the best analytical and extraction methods applicable to this specie and studied compounds. A short screening of its nutritional value in terms of chemical composition is also included. *A. iva* leaves were analyzed for crude protein (CP), cell wall [neutral detergent fiber (NDF), acid detergent fiber (ADF), and acid detergent lignin (ADL)], minerals, fatty acids, essential oils, and phenolic compounds. Mature aerial parts of *A. iva* were randomly collected during the Spring season from Mograne-Zaghouan, Tunisia. Leaves of *A. iva* contained 13.4 ± 0.4% CP, 26.3 ± 0.35% NDF, 20.2 ± 0.42% ADF, and 5.13 ± 0.21% ADL. Mineral content (13.0 ± 0.45%) was mainly composed of potassium (4.5% g DM) and magnesium (4.25% DM). Leaves of *A. iva* had linolenic (26.29 ± 0.760%) and linoleic (37.66 ± 2.35%) acids as the main components of the acid profile. Thymol was found to be the most dominant (23.43%) essential oil, followed by 4-vinylguaiacol (14.27%) and linalool (13.66%). HPLC-PDA-ESI-MS/MS analysis pointed out the presence of phytoecdysteroids. Phenolic acids and flavonoids, such as glycosylated derivatives of naringenin, eriodyctiol, and apigenin, were detected in the methanol extract of *A. iva* leaves. Our results underline the importance of choosing proper extraction methods and solvents to extract and characterize the described compounds profile of *A. iva* leaves. Results also show *A. iva* leaves as a potential source of functional ingredients with beneficial health-promoting properties. Overall, leaves of *A. iva* have low biological activities (antioxidant and antimicrobial activities) with a chemical composition suitable as a feed for ruminants in rangeland pasture. It also has low-grade antibacterial or medicinal characteristics when fed to ruminants.

## 1. Introduction

Determining plants’ biological activity and nutritive values is important before they are included in human or animal diets [1,2]. Also, identifying plant species’ chemical characteristics help to differentiate them from similar species, which often leads to incorrect identification [3]. Therefore, determining nutrient concentrations and amounts of secondary metabolites in plants helps to create databases useful for nutritionists, which results in the accurate inclusion of plant materials that will improve the nutrition of humans and animals, depending on the specific plant’s features.

The extraction method greatly affects the concentration and activity of the secondary metabolites in plants [4] and is the main reason for differences between studies [5]. Extracts with high polarities, such as ethanol, give better results than weakly polar solvents, such as petroleum ether and methanol. Extraction is the foremost step for recovering and isolating phytochemicals from plant materials. Moreover, the concentration of phytochemicals in plants depends on the plant samples’ physical properties and the solvent’s polarity [6]. The extraction efficiency is affected by the chemical nature of phytochemicals, the extraction method, and the solvent used [7,8]. Makni et al. [9] observed that the extraction yield of *A. iva* differed between methanol, aqueous, hexane, and chloroform extractions. Bendif et al. [10] observed different concentrations of total phenolics and free radical-scavenging activity with different extraction methods (acetone, ethanol, and water) of *A. iva*.

The genus *Ajuga* (Labiatae) consists of more than 301 species, mainly found in temperate and warm-temperate zones [11], and many species from this genus are widely used in traditional medicine for the treatment of various illnesses. They have been found to possess hypotensive, hypoglycaemic, vasorelaxant, hypolipidemic, antimicrobial, antifungal, antitumoral, antimalarial, and anthelmintic activities as well as insecticidal and insect antifeedant properties [12,13,14,15,16]. These intriguing biological properties of a member of the genus *Ajuga* were attributable to a wide array of bioactive components, including phytoecdysteroids [14], neo-clerodane diterpenes [15], iridoids [17], sterols and phenols [13].

The species *Ajuga iva* (syn: *A. humilis*; *A. pseudoiva; Teucrium iva*), commonly called “Chandgoura” in Tunisia, Algeria, and Morrocco, has found widespread use in traditional medicine. It is used to treat gastrointestinal disorders, hypertension, hypercholesterolemia, diabetes, fever, toothache, and dysentery [17]. The antioxidant, anti-inflammatory, antimicrobial, insecticidal [14], vasorelaxant, cardiotonic, and wound-healing properties of *A. iva* have also been reported [12,18].

Some studies on *A. iva* have revealed the presence of phytoecdysteroids [4,14], diglycerides, flavonoids [4,18], and essential oils [16]. However, most of these phytochemical studies were focused on the whole or the aerial part of the plant and/or on particular components. Comprehensive phytochemical analysis of the leaves claimed as the most active plant part is undoubtedly lacking. Moreover, it is well known that species belonging to the Labiatae family are characterized by their high intraspecific chemical polymorphism and distinct chemotypes among species from the same genus [19]. Ammar et al. [4] compared the nutritive value of *A. iva* harvested from three different regions of Tunisia, observed differences in the nutritive value of *A. iva* between regions, and recommended season- and region-specific feeding strategies for feeding *A. iva* to animals. Based on that, the main objective of this study was to study the biological activities of *A. iva* in terms of antioxidant and antimicrobial activity of the plant species, together with a short part on its nutritional value in terms of chemical composition. Specifically, this study was designed: (*i*) to determine the mineral content, the fatty acid profile, the chemical composition of the essential oil, and the phenolic constituents of the leaves of the study species; and (*ii*) to evaluate the antioxidant and antimicrobial activities of their different extracts. Such information is a prerequisite to defining the possible areas of application for this species and providing scientific support to its empirical medicinal uses.

## 2. Materials and Methods

### 2.1. Plant Material

Mature aerial parts of *A. iva* were randomly collected in Spring from semi-arid rangeland (average annual precipitations are 440 mm) situated in Mograne-Zaghouan, Tunisia (latitude 36.4302, and the longitude is 10.10276). The area is 140 m above sea level. In order to highlight the nutritional characteristics of *A. iva*, a comparison with alfalfa hay cultivated in the same region was done. The leaves (10 kg) of *A. iva* and samples of alfalfa hay, collected from mature plants, were air-dried at room temperature (40 ± 2 °C) for one week, ground in Retsch blender mill (Normandie-Labo, Normandy, France), and sieved through a 0.5 mm mesh screen to obtain a uniform particle size. The ground substrates were stored in a dark room at room temperature until used.

### 2.2. Chemical Analysis

Dry matter (DM, method ID 934.01), ash (method ID 942.05), and crude protein (CP, method ID 984.13) contents were determined following the methods of AOAC [20]. Total ash content was determined after calcinating 0.5 g in a muffle furnace Manfredi L9C (Manfredi S.R.L, Torino, Italy) at 500 °C for 24 h. The mineral composition was determined after digestion of the sample (500 mg) with 4 mL concentrated nitric acid and 1 mL hydrogen peroxide, using the microwave digestion system Milestone ETHOS 1 (Sorosole, Italy). After cooling at room temperature, the digested samples were diluted up to 25 mL with deionized water for subsequent determination of minerals. Analysis of the elements (Na, K, Ca, Mg, Cu, Fe, and Zn) was carried out only for leaves of *A. iva* by inductively coupled plasma-atomic emission spectrometer, ICP-AES (Horiba Jobin-Yvon Ultima 2 CE). Measurements were performed at the following emission line (nm): Na 589.592, K 769.896, Ca 184.006, Mg 279.553, Cu 324.754, Fe 259.939, and Zn 213.856. The ICP-AES instrumental operating conditions were as follows: Auxiliary gas flow rate 0.5 L/min; RF generator power 1050 W; Plasma gas flow rate 12 L/min; Peristaltic pump’s speed 20 rpm; Pump stabilization time 5 s; Nebulizer flow rate 0.85 L/min.

Analysis of cell wall components in terms of neutral detergent fiber (NDF), acid detergent fiber (ADF), and acid detergent lignin (ADL) was determined with the ANKOM fiber analyzer [21]. Sodium sulfite, but not β-amylase, was added to the solution for the NDF determination.

### 2.3. Total Lipid Content and Fatty Acid Composition

Total lipid extraction was performed according to the AOAC reference method [20], using a Soxhlet apparatus for 8 h with hexane. The final extract was evaporated under reduced pressure in a Heidolph rotary evaporator (Schwabach, Germany), and the total lipids were determined. The defatted raw material was conserved at −20 °C for further analysis.

Prior to analysis by gas chromatography (GC), fatty acids were converted into their corresponding fatty acid methyl esters (FAMEs), using sodium methoxide (3% in methanol) following the method of Cecchi et al. [22]. FAMEs were separated and identified on an Agilent HP 6890 (II) GC equipped with a flame ionization detector (FID) and a polar column TR-FAME (60 m, 0.25 mm i.d., 0.25 µm film thickness). The GC was operated under programmed temperature conditions from 100 °C (5 min) to 240 °C (15 min) at 4 °C/min. The injector and FID detector temperatures were maintained at 240 °C and 206 °C, respectively. Identification of FAMEs was made by comparing their retention time with those of 37 FAMEs Standards (Sigma-Aldrich, Stenheim, Germany). The percent compositions of FAMEs were calculated with reference to the total fatty acids.

### 2.4. Isolation and Analysis of Essential Oils

To isolate *A. iva* essential oils (AIVO), 100 g of ground leaves were submitted to hydrodistillation for 3 h using a Clevenger-type apparatus. The hydrolat was extracted twice with *n*-pentane (20 mL), dried over anhydrous sodium sulfate, then concentrated up to 1.5 mL at 35 °C using a Vigreux column and subsequently analyzed [23]. Analysis of AIVO was performed by gas chromatography-mass spectrometry (GC-MS) using a gas chromatograph HP 6890 interfaced with an HP 5973 mass spectrometer (Agilent Technologies, Palo Alto, CA, USA) with electron impact ionization (70 eV). Separation of the individual compound was achieved by an HP-5MS capillary column (60 m length, 0.25 mm i.d., 0.25 µm film thickness) under the following operating conditions: temperature program: 50 °C (1 min) to 280 °C (8 min) at 5° C/min. Injection temperature: 250 °C; flow rate of the carrier gas helium: 1.2 mL/min; ion source and quadrupole temperatures: 230 and 150 °C, respectively; ionization voltage: 70 eV; mass range: 50–550 u.

The volatile compounds were identified by comparing their retention indices relative to (C_7_-C_20_) *n*-alkanes with the literature and/or with authentic compounds when available. Further identification was made by matching their mass spectra fragmentation patterns with corresponding data (Wiley 275L and NIST05a libraries) and other published mass spectra [24].

### 2.5. Phenolic Compounds Content

#### 2.5.1. Samples Preparation

Five solvents with increasing polarity (petroleum ether, ethyl acetate, ethanol, methanol, and water) were used to prepare different extracts. Dried and ground leaves (1 g) were mixed with 20 mL of solvent in an orbital shaker (150 rpm for 48 h). Each extraction was repeated twice, and the resulting solvent extracts (except for water extract) were filtered through Wattman #1 filter paper (Bärenstein, Germany) and evaporated under reduced pressure in a Heidolph rotary evaporator (Schwabach, Germany). The water extract was frozen and lyophilized in a Christ-Alpha 2–4 freeze drier (Osterode, Germany).

#### 2.5.2. Total Phenolic Content (TPC)

Total phenolics were determined with the Folin-Cieucalteu (FC) assay, according to Lister and Wilson [25]. Briefly, 100 µL of leaf extracts were mixed with 500 µL of freshly diluted 10-fold FC reagent and 1 mL of 20% sodium carbonate solution. After incubation for 1 h in the dark, the absorbance was measured at 760 nm using a Jasco V-630 UV-vis spectrophotometer (Tokyo, Japan). Gallic acid was used as the standard, and results were expressed as micrograms of gallic acid equivalents (µg GAE/g).

#### 2.5.3. Total Flavonoid Content (TFC)

Total flavonoid content was determined by the AlCl_3_ colorimetric method [26]. A 500 µL sample aliquot was mixed with 1.5 mL methanol, 0.1 mL of a 10%AlCl_3_ solution, 0.1 mL of potassium acetate (1 M), and 2.8 mL of distilled water. After 30 min incubation at room temperature, the absorbance was measured at 415 nm. Quercetin was used as a reference standard, and the total flavonoid content was expressed as micrograms of quercetin equivalents (µg QE/g).

#### 2.5.4. HPLC-ESI-MS/MS Analysis

The chromatographic separation and mass spectrometric analyses of phenolics contained in the methanolic extracts were carried out on an Agilent 1100 series HPLC system equipped with a photodiode array detector (PDA) and a triple quadrupole mass spectrometer type Micromass Autospec Ultima Pt interfaced with an ESI ion source. Separation was achieved using a Superspher^®^100 (12.5 cm × 2 mm i.d, 4 µm, Agilent Technologies, Rising Sun, MD, USA) at 40 °C. The samples (20 µL) were eluted through the column with a gradient mobile phase consisting of A (0.1% acetic acid) and B (acetonitrile) with a flow rate of 0.25 mL/min. The following multi-step linear solvent gradient was employed: 0–5 min, 2% B, 5–60 min, 40% B, 60–85 min, 100% B, 85–85.1 min, 2% B, 85.1–100 min, 2% B. PDA detection was performed in the 250–550 nm wavelength range and the mass spectra were recorded in positive ion mode, under the following operating conditions: capillary voltage, 3.2 kV; cone voltage, 40 V; probe temperature, 350 °C; ion source temperature, 120 °C. The spectra were acquired in the *m*/*z* range of 150–750 amu. Tentative identification of phenolic compounds was based on co-chromatography with authentic standards when available. PDA spectra and mass spectra were used to confirm the identity of compounds previously reported in the literature [27,28,29].

### 2.6. Antioxidant Activity

#### 2.6.1. DPPH Radical Scavenging Activity

The DPPH assay followed a reported method [30] with some modifications. Briefly, 1 mL of different extracts was added to 2 mL of a 0.1 mM methanol DPPH solution. The mixture was shaken vigorously and left to stand at room temperature in the dark for 1 h. Thereafter, the absorbance was measured at 515 nm. The scavenging activity was measured as the decrease in absorbance of the samples versus the DPPH standard solution. Results were expressed as radical scavenging activity percentage (%) of the DPPH using the following formula:

% DPPH radical scavenging = [(A_0_ − A_s_)/A_0_] × 100

where A_0_ and A_s_ are the absorbance of the control and the sample, respectively. The effective concentration having a 50% radical inhibition (IC_50_) expressed as mg extract/mL was determined from the graph of the free radical scavenging activity (%) against the extract concentration.

#### 2.6.2. ABTS Scavenging Activity

The ABTS assay was based on the procedure described by Re et al. [31]. The solution consisting of 7 mM of ABTS and 2.4 mM potassium persulfate (1:1 *v*/*v*) was reacted in the dark for 12 h at room temperature. Then it was diluted with methanol to obtain an absorbance of 0.7 at 734 nm. The diluted ABTS solution (2850 µL) was mixed with 150 µL of sample extracts or trolox standard. The mixture was left to stand at room temperature in the dark for 15 min, and then the absorbance was measured at 734 nm. Results were expressed as µmol trolox equivalent (µmol eq. trolox/g), based on the trolox calibration curve.

#### 2.6.3. Ferric Reducing Antioxidant Power (FRAP)

The FRAP assay was carried out as described by Benzie and Strain [32] with slight modifications. Stock solutions of 300 mM acetate buffer, 10 mM TPTZ (2,4,6-tripyridyl-s-triazine) in 40 mM HCl, and 20 mM FeCl_3_. 6H_2_O were prepared. The working FRAP reagent was prepared by mixing the stock solutions in a 10:1:1 ratio. The solution was maintained at 37 °C and pH 3.6. Then, 150 µL of the sample was mixed with 2850 µL of the working solution and left standing at room temperature for 30 min in the dark. The absorbance of the mixture was then measured spectrophotometrically at 593 nm. The change in absorbance was calculated and related to the standard curve generated with trolox. Results were expressed as micromole of trolox equivalent per gram of extract (µmol TE/g).

### 2.7. Antimicrobial Activity

#### 2.7.1. Microbial Strains and Growth Conditions

The different extracts from *A. iva* leaves were individually tested against a panel of 6 microorganisms, including the gram^+^ *Staphylococcus aureus* ATCC8739 and *Streptococcus agalactiae* isolated in the Institute Pasteur (Tunis, Tunisia), the gram^−^ *Enterococcus faecium* ATCC19434, *Salmonella typhimurium* ATCC14028, and *Escherichia coli* ATCC8739, as well as the yeast *Candida albicans* ATCC10231. Microbial strains were cultured overnight at 37 °C in Muller-Hinton agar (MHA). For the antimicrobial screening, the disk diffusion method was employed [33].

#### 2.7.2. Disk Diffusion Method

In this assay, a suspension of the test micro-organisms (10^8^ CFU/mL) was spread on the MHA media plate. The filter paper disk (6 mm in diameter) was soaked with 15 µL of the extracts dissolved in dimethyl sulfoxide (DMSO) and placed on the inoculated plates. After being kept at 4 °C for 2 h, they were incubated at 37 °C for 24 h for bacteria and at 30 °C for 48 h for the yeast. The definite zone of inhibition of any dimension surrounding the paper disk was measured accurately.

## 3. Results

### 3.1. Chemical Composition, Fatty Acid (FA), and Essential Oil Constituents (VOAI) of A. iva Leaves

Data on the chemical composition of leaves of *A. iva* and alfalfa hay is shown in Table 1. As compared with alfalfa hay, *A. iva* leaves contain low total mineral (13.0 ± 0.45%), CP (13.4 ± 0.4%), NDF (20.2 ± 0.42%) and ADF (20.2 ± 0.42%).

However low lipid contents (2.18 ± 0.58%) were detected in the leaves of *A. iva*. The ICP-AES analysis (Table 2) allowed the detection of 7 major elements from which K was the most dominant (3201.0 ± 143.88 mg/kg DM) and Cu the lowest (87.9 ± 3.34 mg/kg DM).

Analytical GC analysis showed that unsaturated fatty acids (UFA) contributed more than 89% of the total fatty acid profile (Table 3). Linolenic (37.66%), linoleic (26.29%), and oleic (15.91%) were the most abundant UFA. Palmitic (16.82%) and stearic (2.18%) acids were the principal saturated fatty acids (SFA).

The chemical composition of AIVO was determined by GC-MS analysis. The essential oil compositions, along with their retention index and percentage composition, are listed in Table 4. Within the 21 identified volatile compounds, thymol was found to be the most dominant (23.43%), followed by 4-vinylguaiacol (14.27%) and linalool (13.66%) (Table 4).

### 3.2. Extract Yield, Total Phenolic, Flavonoid Contents of Different Solvent Extracts

A total of five solvents (petroleum ether, ethyl acetate, ethanol, methanol, and water) were used to extract polyphenolic compounds from *A. iva*. As shown in Table 5, depending on extraction solvent, the extract yield ranged from 0.82 ± 0.27% (petroleum ether) to 12.85% ± 1.40% (water). The contents of phenolic compounds were variable, with maximum levels found in methanol (177 GAE/g extract) followed by aqueous (144 GAE/g extract) and ethanol (143 GAE/g extract) extracts. On the other hand, total flavonoids assumed maximum amounts in ethyl acetate extract (52.61 QE/g extract). An intermediate value was revealed in methanol extracts (40.23 QE/g extract).

### 3.3. HPLC-PDA-MS/MS Analysis of the Methanol Extract

**Figure 1:**
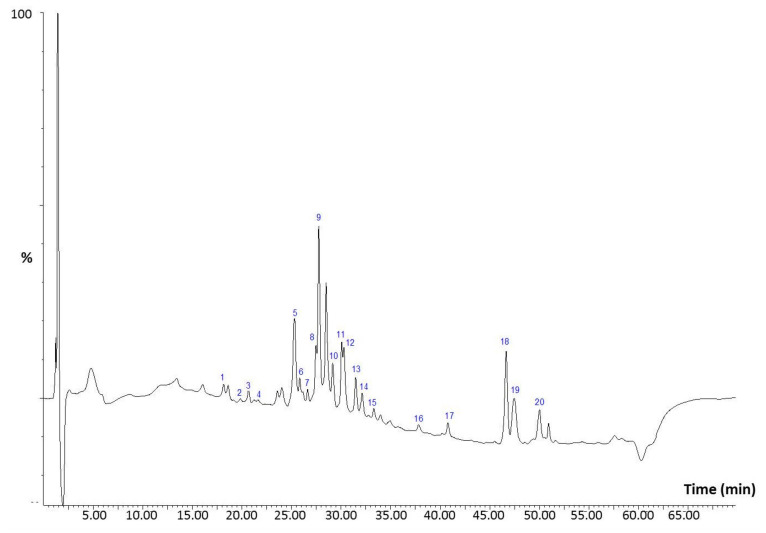
Total Ion Chromatogram (TIC) of the methanol extract of *A. iva* leaves.

The total ion chromatogram of the methanol extract of *A. iva* is shown in Figure 1, and the peak characteristics as well as tentative identification are summarized in Table 6. Twenty compounds were tentatively identified based on their UV and mass spectral data as well as by comparison of their retention time (RT) with those of available commercial standards. As expected, the main components showed a maximum absorbance of around 247 nm which represents the UV spectral characteristic of phytoecdysteroids (peaks 1, 7, 9, 11, 12, 16, and 20), typical components of the genus *Ajuga*. Peak 1 (*tr* = 18.15 min; λmax of 237 nm) showed a pseudomolecular ion [M-H]^−^ at *m/z* 495 corresponding to polypodine B. Peak 7 (*tr* = 26.62 min) gave a deprotonated molecular ion [M-H]^−^ at *m/z* 463 indicative to β-ecdysone. Peak 9 (*tr* = 27.75 min) exhibited an [M-H]^−^ ion at *m/z* 539 by forming an adduct with acetic acid and the main fragment ion [M-H]^−^ at *m/z* 479 consistent with 20-hydroxyecdysone. Peak 11 (*tr* = 30.06 min) showed a precursor ion at *m/z* 553 [M-H + acetic acid adduct]^−^ and its MS^2^ spectrum produced a fragment ion [M-H]^−^ at *m/z* 493 which is characteristic of Makisterone A. Peak 12 (*tr* = 30.28 min) was tentatively identified as cyasterone based on the [M-H + acetic acid adduct]^−^ at *m/z* 579 and the product ion at 519. Peak 16 (*tr* = 37.83 min) gave a deprotonated molecular ion at *m/z* 577 [M-H + acetic acid adduct]^−^ and the main fragment ion [M-H]^−^ at *m/z* 517 and was tentatively identified as 24-dehydroprecyasterone. Peak 20 (*tr* = 50.02 min) with a pseudo molecular ion [M-H]^−^ at *m/z* 481 was tentatively identified as 7,8-dihydroajugasterone C.

Peak 18 eluted at 46.64 min, showed a characteristic UV absorption band for sterols (λ_max_ 208 nm) and a pseudomolecular [M-H]^−^ ion at *m/z* 577 similar to that observed for campestanol *trans*-ferulate. Peak 19 (*tr* = 47.45 min) had similar absorbance to peak 18 indicative of a sterol component and exhibited a deprotonated [M-H]^−^ ion at *m/z* 329 and the main fragment at *m/z* 311 [M-H-18] (corresponding to the loss of H_2_O molecule). The observed fragmentation pattern was not informative enough to elucidate the structure of this sterol.

In addition to the aforementioned sterols and phytoecdysteroids, other components including phenolic acids and flavonoids were also identified. Peak 2 (*tr* = 19.87 min; λ_max_ 225–303 nm) had a pseudo molecular [M-H]^−^ ion at *m/z* 325 and yielded the main fragment at *m/z* 163 (indicative of the aglycone coumaric acid) through a loss of hexose residue (162 amu). This compound was identified as *p*-coumaroyl hexose. Peak 3 (*tr* = 20.60 min; λ_max_ 279 nm) showed a deprotonated molecular [M-H]^−^ ion at *m/z* 457 releasing a fragment ion at *m/z* 305 (indicative of epicatechin gallate) through the loss of galloyl residue (152 amu) was assigned as epigallocatechin gallate.

Peak 4 (*tr* = 21.23 min; λ_max_ 245, 331 nm) produced a deprotonated molecular [M-H]^−^ ion at *m/z* 449 and an eriodyctiol MS^2^ fragment at *m/z* 287 following the loss of hexoside residue (162 amu), which indicates the presence of eriodyctiol-7-hexoside. Peak 5 (*tr* = 25.04 min; λ_max_ 271, 331 nm) with a pseudomolecular [M-H]^−^ ion at *m/z* 593, which in turn produced a major fragment ion at *m/z* 269 (apigenin aglycone) through the loss of di-hexose moiety (324 amu) was proposed as apigenin-dihexoside. Peak 6 (*tr* = 25.82 min; λ_max_ 253, 349 nm) with a pseudomolecular [M-H]^−^ ion at *m/z* 607 and MS^2^ fragment at *m/z* 299 diosmetin aglycone through the loss of neohesperidoside residue ([M-H-308]) was tentatively identified as diosmetin-7-*O*-neohesperidoside. Peak 8 (*tr* = 27.44 min; λ_max_ 342 nm) displayed a similar fragmentation pattern to the standard chrysoeriol. Peak 10 (*tr* = 29.15 min; λ_max_ 323 nm) with [M-H]^−^ ion at *m/z* 301 was positively identified as ellagic acid by comparison with the UV and mass spectra of the authentic standard.

Peaks 13, 14, and 15 were identified as naringenin derivatives (λ_max_ 271, 329 nm) derivatives. In fact, peak 13 eluted at 31.47 min exhibited a pseudomolecular [M-H]^−^ ion at *m/z* 579 and the main fragment at *m/z* 271 (naringenin aglycone) through the loss of rutinoside moiety (308 amu) was assigned to naringenin-7-*O*-rutinoside. Peak 14 (*tr* = 32.12 min) exhibited a similar fragmentation pattern to the authentic standard naringenin, consequently, it was unequivocally identified as naringenin. Peak 15 was an isomer of peak 13 ([M-H]^−^ ion at *m/z* 579 and the main fragment at *m/z* 271) and was tentatively identified as naringenin-7-O-neohesperidoside.

### 3.4. Antioxidant Activity

The screening of the antioxidant capacity of the methanolic extracts was evaluated by three methods, namely the DPPH, ABTS radical scavenging capacity, and the ferric reducing antioxidant power (FRAP) (Table 7). A significant variation was observed between extracts, regarding their ability to scavenge DPPH•. The IC_50_ values of the DPPH radical scavenger ranged from 411.83 to 3328.42 μg/mL for the five extracts tested, with the highest scavenging capacity for water and methanol (IC50 411.83–729.98 µg/mL). The same trend was found in the ABTS test (1161.52–148.51 µg/mL). Moreover, the most important reducing power was interestingly observed also in aqueous and methanol extract (0.12–0.13 TE/g extract). However, they were by far less effective than the synthetic antioxidant trolox.

### 3.5. Antimicrobial Activity

The in vitro antimicrobial activity of the *A. iva* extracts was qualitatively evaluated by the presence or absence of an inhibition zone (IZ). As can be seen in Table 8, the most susceptible strain was found to be *S. agalactiae* (Gram^+^) which was particularly sensitive to the most of extracts with methanol, ethanol, and water is the most active. The Gram^+^ *E. faecium*, the Gram- S. typhimurium, and *E. coli* were also found to be sensitive to most of the extracts with the highest inhibition observed with ethanol extract. The latter extract showed weak activity against the yeast *C. albicans*. All tested microorganisms were more susceptible to Ampicillin (20–44 mm) than to the extracts.

## 4. Discussion

It is pertinent to mention that the nutritional value of *A. iva* cultivated from different Tunisian areas was earlier studied [4]. According to these authors, cultivar collected from Mograne had the lowest cell wall contents and the highest digestibility. Therefore, this cultivar collected from the Mograne area was the subject of the determination of biological activities reported in this current research. On the other hand, it is worth remembering that our main objective is to present complementary data to the nutritional value of the most appropriate cultivar, as animal fodder, on its medicinal characteristics in terms of antioxidant and antimicrobial activity, when different solvents are studied. Crude protein content (13.4%) is comparable to that of alfalfa hay and is considered enough to meet the minimum CP requirement (8% of DM) for optimal microbial function and the maintenance of adequate ruminal fibrolytic activity [6,34]. In this same context, Sampaio et al. [34] reported that increasing the dietary CP concentration close to 10% DM was found to optimize the use of low-quality forage and considered supplying enough nitrogen to maintain the microbial activity in the rumen. The low ADL content (5.1%) of *A. iva* is an indicator of a low lignification of its cell wall and thus a high digestibility is expected.

On the other hand, high values in mineral content (>10%) seem to be characteristic of the Lamiaceae family. This result was consistent with those reported for the genus *Ocimum*, *Perilla*, *Hyptis*, *Scuttelaria*, *Mentha,* and *Nepeta* [35,36,37]. As mentioned in Table 2, the ICP-AES analysis allowed detection of 7 major elements with the following trend: K > Mg > Ca > Na > Fe > Zn > Cu.

To the best of our knowledge, this is the first report on the mineral composition of *A. iva*. Nevertheless, the only chemical investigation of *A. bracteosa* revealed that Ca, Mg, K, and Na were the main macro-elements, while Fe, Cr, Mn, Cu, and Zn were the major microelements [38]. Collectively, these results indicate that *A. iva* leaves could be considered an excellent source of essential minerals and encourage its use in diet and/or dietary supplement. The abundance of K, and to a less extent Mg, Ca and Fe was expected if we bear in mind that these elements have structural and functional roles in plants [39].

From a medicinal point of view, the presence of some essential minerals which have pivotal physiological and biochemical roles in the human body (e.g., enzymatic reactions, energy production, and transmission of nerve impulses, among others) could justify the traditional medicinal uses of *A. iva.* Support for this assumption was given by the low value of the Na/K ratio, suggesting the potential of *A. iva* leaves for managing hypertension. Additionally, it has been reported that incorporation of Mg-rich extract or Zn-rich extracts of *Atriplex balimus* in the diet was associated with better insulin release, high uptake of glucose by fat cells, and reduced arterial pressure, with the effects being modulated by the presence of K and Ca [40]. The lipid-lowering properties and anti-obesity effects of Zn and Ca have also been reported [41].

As mentioned before, the average total lipid content was 2.18% (*w*/*w*) (Table 1). This value was lower than those reported for *A. reptans* L. (13.14%), *A. chia* (Poir) Schreb (12.03%), and *A. genevensis* L. (10.20%) [42]. However, it was 3.2-fold higher than the total lipid content in the aerial part of *A. turkestanica* (Rgl.) Briq [43]. These low levels would reflect the low essential oil contents of *A. iva*.

The fatty acid profile (C18:3 > C18:2 > C16:0 > C18:1) differed from those reported in *A. reptans*, *A. chia*, and *A. genevensis* where the following profile was obtained C18:2 > C18:3 > C18:1 > C16:0 [42]. Studies reported by Khidoyatova et al. [43] revealed that linolenic, oleic, linoleic, and palmitic acids were found to be the main components of neutral lipids, glycolipids, and phospholipids of *A. turkestanica* aerial parts. However, the profile C16:0 > C18:2 > C18:1 was found in *A. genevensis* and *A. chia* [44]. Such discrepancies are likely due to genetic and environmental factors [45]. Moreover, given the importance of polyunsaturated fatty acids (i.e., linolenic and linoleic acids) as health-promoting nutrients and their potential in alleviating cardiovascular, inflammatory, heart, and neurodegenerative diseases, as well as atherosclerosis, autoimmune disorder, and diabetes [46], the high amounts of the essential linolenic and linoleic acids, makes the lipids of *A. iva* leaves important for nutritional applications and support their use as a food supplement.

The chemical composition of AIVO revealed the presence of 21 compounds covering more than 91% of the GC profile (Table 3). Similarly, results reported by Chouitah et al. [47] on *A. iva* collected from Algeria identified 22 compounds with a cover rate of 99.92%. However, studies reported by Bouyahya et al. [48] pointed out the presence of 28 volatile compounds in essential oils of *A. iva* harvested at different phonological stages (vegetative, flowering, and post-flowering stages) from Morocco. It appears, therefore, that some factors, such as the origin and plant growing environment [19] and phenological stage [48] regulate the secretion of essential oils.

On the other hand, it is pertinent to notice that the identified essential oils belonged to five different groups classified according to the importance of their presence as follows: phenol (39.53%) > alkane (28.65%) > oxygenated monoterpen (16.86%) > alcohol (4.78%) > phenylpropen (2.1%). The phenol group was mostly represented by thymol (23.43%) and the alkane group was represented by heptacosane (4.14%) and hexacosane (3.96%). Linalool was the main component (13.66%) of the oxygenated monoterpene group, while octen-3-ol was the chief component (4.02%) of the alcohol group. Phenylpropenes were represented by eugenol (2.1%). Studies reported by Bouyahya et al. [48] pointed out that, at the vegetative stage of *A. iva*, three major essential oils were identified, namely carvacrol (32.72%), octadecane (17.28%) and methyl chavicol (8.18%); while thymol (1.25%) and linalool (1.17%) were less abundant, as compared by those reported in our present study. Moreover, these same authors revealed that the most dominant group was oxygenated monoterpen (62.24%), which was far higher than its presence in our study. Chemical composition of two varieties of *A. chamaepitys* subsp. *chia* var. *chia* and var. *ciliate* studied by Baser et al. [49] revealed that β-pinene (20.8%) and germacrene D (12.6%) were the main components in the former variety, while germacrene D (14.6%) and β-pinene (14%) were dominants in the latter variety. In this same context studies reported by Azizan et al. [50] on *A.* *chamaepitys* growing in Iran reported that β-pinene and α-pinene are by far the dominant compounds (34.3% and 16.1%, respectively). These same authors pointed out that other compounds present in rather appreciable amounts were the monoterpene hydrocarbons y-terpinene (7.7%), limonene (6.1%), and mycrene (1.4%).

In another report from the same country and same variety, *p*-cymene (34.5%), β-pinene (18%), α-phellandrene (17.8%), and α-pinene (15.2%) were described as the main component of *A. chamaecistus* [51]. Contrarily, the essential oils of *A. orientalis* from Iran were found to be rich in germacrene D (24.2%), β-cubebene (18.3%), and β-caryophyllene (16.9%) [52]. More recently, the chemical composition of the essential oil of *A. parviflora* from India has been described with β-caryophyllene (22.4%), γ-muurolene (12.7%), γ-terpinene (6.3%), caryophyllene oxide (6.2%) and α-humulene (5.8%) were the main components [53]. It appears therefore that the differences in chemical components of AIVO would be attributed to the fact that aromatic plants metabolize volatile compounds by genetic profiles, planting area, and environmental factors [19].

Yields of different extracts obtained from leaves of Tunisian *Ajuga iva* varied significantly. The highest extract yield was obtained by methanol extraction. The petroleum ether efficiency as a solvent was lower than the efficiencies of all other solvents. This finding is in accordance with some studies which demonstrate that maximum extract yield, was obtained with methanol and water [9]. The extraction yield appears to be influenced by the polarity degree of the solvent, and by the degree of the polarity of the various extract components such as phenolic constituents [54]. It has been also reported that the efficiency of the extraction depends on the extraction time and temperature [55].

Furthermore, the contents of phenolic compounds of *Ajuga iva* varied according to the solvent used for the extraction process. Results revealed that methanol extract had the highest contents of total phenols. Our results were higher than those obtained in the same species from Morocco [5,56] and Algeria [55,57,58]. These variations may be due to many factors, including geographical, genetic, and ecological factors [59]. The lower polarity solvents, particularly: Petroleum Ether, and ethyl acetate showed much lower ability in extracting the phenolic compounds as compared to the polar solvents. In contrast, ethyl acetate extracts had more condensed flavonoids than the remaining extracts. These results were in line with those reported by Anokwuru et al. [60] and Senhaji et al. [5] where ethyl acetate was found to be the best solvent for the extraction of flavonoids. So, solvent polarity will play a key role in increasing phenolic solubility [61]. Besides, the solubility of phenolic compounds is governed by the type of solvent used, the degree of polymerization of phenolics, and their interaction [55].

Prompted by these results showing high total phenolic content in methanol extracts, we decided to analyze the phenolic composition to get a deeper insight into specific components of the polyphenolic mixture.

The HPLC-ESI-MS/MS allowed for the identification of twenty compounds in *A. iva* methanol extracts, including phytoecdysteroids (polypodine B [29], β-ecdysone [62], 20-hydroxyecdysone [28], makisterone A [29], cyasterone [63], 24-dehydroprecyasterone, 7,8-dihydroajugasterone C [64]), sterols (campestanol *trans*-ferulate [27] and phenolic acids and flavonoids (*p*-coumaroyl hexose, epigallocatechin gallate, eriodyctiol-7-hexoside, apigenin-dihexoside, diosmetin-7-*O*-neohesperidoside [65], chrysoeriol, ellagic acid, naringenin-7-*O*-rutinoside, naringenin, naringenin-7-*O*-neohesperidoside).

Many reports have been published on the chemical composition of *A. iva*, mentioning the evidence of numerous bioactive compounds, such as neo-clerodanediterpenoids, phytoecdysteroids, tannins, and iridoids [66]. Our results revealed that 20-hydroxyecdysone was the main component identified in the extracts. Moreover, high levels of campestanol *trans*-ferulate were found. This is consistent with the general abundance of *Ajuga* plants in phytoecdysteroids 20-hydroxyecdysone [67,68].

Among the revealed flavonoids, apigenin-dihexoside is the predominant one. In line with that, Boudjelal et al. [66] revealed that two flavonoids appeared to predominate, apigenin. and naringenin, in the aqueous infusion of *A. iva*. However, Khattelia et al. [69] reported the presence of caffeic acid, *p*-coumaric acid, rutin, and luteolin in the extract of the aerial part of *A. iva*.

Regarding the antioxidant activity, it can be inferred that total phenolic content may be indicative of the antioxidant capacity of *A. iva* leaves. In fact, polar extracts namely water and methanol were found as the most effective antioxidant in the DPPH, ABTS, and FRAP assays. Less promising results were revealed by Sönmez et al. [70] for methanol extracts of *A. postii* and *A. relicta* species. Compared to results reported on *A. iva* from Morocco, the Tunisian species exhibited less activity [5]. Differences in the antioxidant activity could be explained by the presence of some phenolic compounds that may enhance the antioxidant capacity of extracts [71]. In fact, Mamadaliva et al. [72] revealed that the low antioxidant activity of *A. turkestanica* extracts was attributed to the presence of phytoecdysteroids, recognized for their weak antioxidant activity [72]. However, major and minor components as well as the synergetic or antagonistic interactions between constituents could give rise to the exhibited biological activities. The presence of phytoecdysteroids makes *A. iva* suitable for other applications including hypoglycaemic [73], antidiabetic [18], hypolipidemic [74], and wound healing [75], among others.

The antimicrobial activity estimated by the diameter of inhibition varied according to extracts and microbial strains. The inhibition zones ranged between 8 and 15 mm. The antimicrobial activity of all extracts depends largely upon the concentration of extracts, the bacterial strains, and the type of solvent. The ethanol extract was slightly more effective than the remaining extracts. In line with our results, Toiu et al. [76] revealed that the bacterial strains were more sensitive to the ethanol extract of *A. laxmannii*. The most active antimicrobial compounds are rather extracted by ethanol. In fact, ethanol extracts contain higher amounts of iridoids which had a wide range of biological and pharmacological activities [71]. However, our results are contradictory to those reported by Makni et al. [9] who reported strong antimicrobial activity against *E. coli*, *S. aureus*, and *Fusarium* Sp. of the methanol extract of *A. iva*. These discrepancies might be linked to differences in the sample preparation and bioassays procedures.

Overall, this study’s results provide a baseline for assessing the biological activities of *A. iva* (i.e., the antioxidant and antimicrobial activities) of *A. iva* and the nutritional value in terms of chemical composition. Moreover, such results help to catalog other fodder species such as alfalfa. Hence, the introduction of *A. iva* becomes a valuable addition to the available information as this plant has high nutraceutical value. Therefore, based on previous studies [4] and data from the present study, further research should consider feeding *A. iva* leaves to different ruminants and determine their effects on nutrient degradability and animal’s productive performance. Then, the next step could be to test *A. iva* leaves in on-farm conditions.

## 5. Conclusions

This study was carried out to highlight the dual characteristics of leaves of Tunisian *A. iva*, in terms of nutritional value and biochemical characteristics (antioxidant and antimicrobial activities) and the main essential oils using different extracts. Based on its reasonable CP content (>13% DM) and the low lignification of the cell wall, leaves of *A. iva* collected from the Mograne area can be considered as a feed for ruminants as an alternative to common forages such as alfalfa (~13% CP). The contents of phenolic compounds varied between extractors, with maximum levels found in methanol followed by aqueous and ethanol extracts, demonstrating the most suitable extractors to use when doing analytical analysis. The analytical data showed that phytoecdysteroid, 20-hydroxyecdysone, was the most abundant compound in *A. iva* methanol extracts. The presence of these components recognized for their low antioxidant and antimicrobial activities could justify the weak biological activities obtained herein. Despite that, leaves of *A. iva* might represent an excellent source of functional food ingredients that could be used in food and pharmaceutical applications as a medicinal plant. Further studies need to evaluate how the low-grade antimicrobial effects and the higher polar extracts’ effectiveness (especially aqueous) of *A. iva* leaves will affect rumen microflora activity, and how different rumen microorganisms and pathways (i.e., methanogenesis, protein synthesis) are affected by diverse rates of inclusion in the diet. Hence researchers should consider feeding *A. iva* leaves to different ruminant species and determine their effects on nutrient degradability and animal productive performance.

## Figures and Tables

**Table 1:** Chemical composition (% DM) of leaves of *A. iva* and alfalfa hay collected from rangelands of Mograne (*n* = 3 samples, 3 replicates per sample).

Item	Mean ± SD	
	*A. iva*	Alfalfa Hay
Dry matter	89.8 ± 0.44	90.5 ± 0.75
Ash	13.0 ± 0.45	21.8 ± 0.15
Crude protein	13.4 ± 0.40	15.2 ± 0.23
Neutral detergent fiber	26.3 ± 0.35	57.9 ± 0.62
Acid detergent fiber	20.2 ± 0.42	46.2 ± 0.77
Acid detergent lignin	5.13 ± 0.21	13.5 ± 0.65
Ether extract	2.18 ± 0.58	1.05 ± 0.15

**Table 2:** Ash content and mineral composition of *A. iva* leaves (n = 3 samples, 3 replicates per sample).

Item	Mean ± SD
Ash content (%, DM)	13.0 ± 0.45
Minerals (mg/kg dry weight)	
Potassium (K)	45,035.7 ± 327.51
Magnesium (Mg)	4255.4 ± 113.69
Calcium (Ca)	3201.0 ± 143.88
Sodium (Na)	1607.7 ± 53.24
Iron (Fe)	1031.4 ± 72.37
Zinc (Zn)	413.8 ± 32.24
Copper (Cu)	87.9 ± 3.34
Na/K ratio	0.037 ± 0.001
Zn/Cu	4.71 ± 0.02

**Table 3:** Total lipid content and fatty acid profile of *A. iva* leaves (*n* = 3 samples, 3 replicates per sample).

Item	Mean ± SD
Total lipid content (% DM)	2.18 ± 0.58
Fatty acids (% total fatty acids)	
Palmitic (C16:0)	16.82 ± 0.48
Palmitoleic (C16:1)	0.6 ± 0.02
Stearic (C18:0)	2.18 ± 0.06
Oleic (C18:1)	15.91 ± 1.28
Linoleic (C18:2)	26.29 ± 0.76
Linolenic (C18:3)	37.66 ± 2.35
Arachidonic (C20:0)	0.54 ± 0.02
Saturated Fatty Acids (SFA)	19.54 ± 1.22
Unsaturated Fatty acids (UFA)	80.46 ± 2.64
UFA/SFA ratio	4.11
n-6/n-3 ratio	0.69

**Table 4:** Essential oil composition (% total peak area) of *A. iva* leaves (*n* = 3 samples, 3 replicates per sample).

Compound	RI ^1^	(%)
Z-3-Hexenol	858	0.76
Octene-3-ol	978	4.02
Linalool	1088	13.66
*trans*-pinocarveol	1139	1.61
α-terpineol	1190	1.59
Thymol	1266	23.43
Carvacrol	1277	1.83
*p*-vinylguaiacol	1315	14.27
Eugenol	1356	2.1
Docosane	2200	0.55
Tricosane	2300	1.06
Tetracosane	2400	2.26
Pentacosane	2500	3.13
Hexacosane	2600	3.96
Heptacosane	2700	4.14
Octacosane	2800	3.93
Nonacosane	2900	3.36
Triacontane	3000	2.61
Hentriacontane	3100	1.77
Dotriacontane	3200	1.2
Tritriacontane	3300	0.68

^1^ RI: retention index on HP-5MS column.

**Table 5:** Effects of extracting solvent on extract yield, total phenolic and flavonoid contents (*n* = 3 samples, 3 replicates per sample).

Solvent	Extract Yield (%) ^1^	TPC (mg GAE/mg DM) ^2^	TFC (mg CE/mg DM) ^3^
Petroleum Ether	0.82 ± 0.28 d	95.25 ± 0.59 d	28.28 ± 0.28 c
Ethyl acetate	2.8 ± 0.31 c	119.02 ± 0.91 c	52.61 ± 0.86 a
Ethanol	9.68 ± 0.62 b	143.66 ± 0.72 b	19.02 ± 0.31 d
Methanol	8.56 ± 0.42 b	177.74 ± 0.44 a	40.23 ± 0.39 b
Aqueous	12.85 ± 1.40 a	144.11 ± 0.53 b	30.88 ± 0.89 c

Superscript within the same column, mean values not sharing a common superscript represent significant differences (*p* < 0.05). ^1^ Extract yield expressed as percentage dry weight. ^2^ Total phenol content (TPC) expressed as mg GAE/g extract. ^3^ Total flavonoid content (TFC) expressed as mg QE/g extract.

**Table 6:** Retention time, maximum (RT), UV absorption (λ_max_), mass spectral data, and tentative identification of the phenolic compounds in the methanol extract of *A. iva* leaves (n = 3 samples, 3 replicates per sample).

N°	RT (min)	UV (λ Max)	[M-H]^−^	Main Fragment	Tentative Identification	%
1	18.15	237	495		Polypodine B	1.55
2	19.87	225.303	325	163	*p*-coumaroyl hexose	0.29
3	20.60	279	457	305	Epigallocatechin gallate	1.21
4	21.23	245.331	449	287	Eriodyctiol-7-hexoside	0.37
5	25.04	271. 331	593	269	Apigenin-dihexoside	9.33
6	25.82	253.349	607	299	Diosmetin-7-*O*-neohesperidoside	1.12
7	26.62	247	463		β-ecdysone	0.8
8	27.44	342	299		Chrysoeriol	3.23
9	27.75	247	479		20-hydroxyecdysone	14.63
10	29.15	269.323	301		Ellagic acid	3
11	30.06	247	493		Makisterone A	4.92
12	30.28	247	519		Cyasterone	5.82
13	31.47	271.329	579	271	Naringenin-7-*O*-rutinoside	3.13
14	32.12	271.327	271		Naringenin	2.08
15	33.3	269.329	579	271	Naringenin-7-*O*-neohesperidoside	0.95
16	37.83	247	517		24-dehydroprecyasterone	0.88
17	40.78	197	363		Harpagide	1.23
18	46.64	208	577		Campestanol *trans* ferulate	10.5
19	47.45	208	311		Unknown sterol	6.85
20	50.02	247	481		7,8-dihydroajugasterone C	4.37

**Table 7:** Antioxidant activity (DPPH, ABTS, and FRAP assay) of *A. iva* extracts (*n* = 3 samples, 3 replicates per sample).

Solvent	DPPH ^1^	ABTS	FRAP ^2^
Petroleum Ether	3328.42 ± 11.89 ^a^	5216.96 ± 38.96 ^b^	0.03 ± 0.00 ^c^
Ethyl acetate	1461.07 ± 6.09 ^c^	4654.90 ± 51.67 ^c^	0.07 ± 0.01 ^b^
Ethanol	2535.07 ± 9.26 ^b^	7447.82 ± 34.13 ^a^	0.02 ± 0.00 ^c^
Methanol	411.83 ± 8.60 ^e^	1161.52 ± 23.30 ^e^	0.13 ± 0.01 ^a^
Aqueous	729.98 ± 7.21 ^d^	1485.51 ± 36.18 ^d^	0.12 ± 0.01 ^a^

Superscript (a, b, c, d, e) within the same column, mean values not sharing a common superscript represent significant differences (*p* < 0.05). ^1^ DPPH: IC_50_, the scavenging of the free anionic 2,2-diphenyl-1-picrylhydrazyl radical expressed in µg/mL. ^2^ FRAP, ferric reducing antioxidant power value expressed as mM TE/g extract.

**Table 8:** Antimicrobial activity (expressed as the diameter of zone of inhibition) of different extracts of *A. iva* leaves (*n* = 3 samples, 3 replicates per sample).

	Gram-Positive Bacteria		Gram-Negative Bacteria			Yeast
Extracts	*Staphylococcus aureus*	*Streptococcus agalactiae*	*Enterococcus faecium*	*Salmonella typhimurium*	*Escherichia coli*	*Candida albicans*
Petroleum ether	NA	NA	NA	NA	NA	NA *
Ethyl acetate	NA	10	NA	8	10	NA
Ethanol	NA	14	15	9	11	11
Methanol	NA	14	8	8.5	10	NA
Water	10	12	12	9	9	11
Ampicillin	40	38	44	20	14	40

* NA, not active.

## Data Availability

The data presented in this study are available on request from the corresponding author.
